# Intersegment Recombination During Influenza A Virus Replication Gives Rise to a Novel Class of Defective Viral Genomes

**DOI:** 10.3390/v17060856

**Published:** 2025-06-16

**Authors:** Soraya Anisi, George Noble, Rory Williams, Jack Hales, Hannah E. Bridgewater, Andrew Easton, William Collier, Phillip Gould

**Affiliations:** 1 Centre for Health and Life Sciences, Coventry University, Coventry CV1 2UD, UK; anisis2@coventry.ac.uk (S.A.); nobleg@coventry.ac.uk (G.N.); william31@coventry.ac.uk (R.W.); h.bridgewater.1@warwick.ac.uk (H.E.B.); 2 OVO Biomanufacturing Ltd., Friars House, Manor House Drive, Coventry CV1 2TE, UK; j.hales@ovobiomf.com (J.H.); w.collier@ovobiomf.com (W.C.); 3 School of Life Sciences, University of Warwick, Coventry CV4 7AL, UK; a.j.easton@warwick.ac.uk; 4 College of Engineering, Environment and Science, Coventry University, Coventry CV1 2JH, UK

**Keywords:** influenza A virus, defective viral genomes, RNA recombination, next-generation sequencing, multisegment recombination, viral evolution

## Abstract

Influenza A virus (IAV) is a highly diverse pathogen with genetic variability primarily driven by mutation and reassortment. Using next-generation sequencing (NGS), we characterised defective viral genomes (DVGs) generated during the serial passaging of influenza A/Puerto Rico/8/1934 (H1N1) virus in embryonated chicken eggs. Deletions were the most abundant DVG type, predominantly accumulating in the polymerase-encoding segments. Notably, we identified and validated a novel class of multisegment DVGs arising from intersegment recombination events, providing evidence that the IAV RNA polymerase can detach from one genomic template and resume synthesis on another. Multisegment recombination primarily involved segments 1–3 but also occurred between other segment pairings. In specific lineages, certain multisegment DVGs reached high frequencies and persisted through multiple passages, suggesting they are not transient by-products of recombination but may possess features that support stable maintenance. Furthermore, multisegment DVGs were shown to be encapsidated within virions, similar to deletion DVGs. The observation of recombination between segments with limited sequence homology underscores the potential for complex recombination to expand IAV genetic diversity. These findings suggest recombination-driven DVGs represent a previously underappreciated mechanism in influenza virus evolution.

## 1. Introduction

Influenza A virus (IAV) is a major respiratory pathogen that causes seasonal epidemics and sporadic pandemics, leading to significant global morbidity and mortality [1]. Annual influenza epidemics are estimated to result in 290,000 to 650,000 deaths, with the greatest risk of severe disease seen in young children, the elderly, and immunocompromised individuals [2,3,4]. While antiviral treatments have had a limited impact, seasonal vaccination remains the most effective strategy for reducing disease burden [5,6].

Defective viral genomes (DVGs) are naturally occurring, non-infectious variants of wild-type viral genomes that arise from errors during replication [7,8,9]. Although DVGs retain the essential 5′ and 3′ terminal sequences required for replication, they often harbour large internal deletions, insertions, or sequence rearrangements that disrupt protein-coding potential [10,11]. DVGs cannot replicate independently and require co-infection with a helper virus that supplies the missing viral components. When incorporated into new virions, they form defective interfering particles (DIPs), facilitating their amplification and persistence within the viral population [12,13].

DVGs have been identified in a wide range of positive- and negative-sense RNA viruses, including Flock House virus, coronaviruses, and influenza viruses [14,15,16]. In these viruses, DVGs can modulate infection through two principal mechanisms: interference with the replication and/or packaging of the wild-type virus genome and activation of innate immunity. DVGs compete with full-length genomes for viral polymerase, replication machinery, and packaging, thereby limiting the production of infectious wild-type progeny [17]. Additionally, DVGs are potent stimulators of the innate immune system, such as RIG-I, triggering strong type I interferon (IFN) responses that enhance antiviral immunity [18,19]. Due to their ability to suppress viral replication and enhance host immunity, DVGs are being investigated both as potential antiviral therapeutics for influenza and other respiratory viruses and as agents that may negatively impact vaccine yield [12,20,21]. A comprehensive understanding of naturally occurring DVG populations is therefore essential for elucidating the evolutionary dynamics of IAV and informing novel strategies to reduce the disease burden.

Illumina short-read sequencing, with its high throughput and ~99% base accuracy, provides a powerful next-generation sequencing (NGS) platform for detecting low-frequency DVG variants and precisely resolving recombination breakpoints at single-nucleotide resolution [22,23]. Unlike targeted PCR-based methods, Illumina sequencing is not constrained by primer design, enabling an unbiased and comprehensive view of DVG populations [19]. Several bioinformatics pipelines have been developed to identify and characterise DVG junctions, including ViReMa [24], DI-tector [25], and DVG-profiler [26]. Among these, ViReMa is a widely used open-source tool that has been used across multiple RNA viruses, including IAV [15,23,27].

While most studies on IAV DVGs have focused on characterising intra-segmental deletion variants [10,28], emerging evidence indicates that the spectrum of DVG diversity extends beyond simple deletion events. In other RNA viruses, DVGs containing insertions and multisegment recombination events have been identified, suggesting that DVG formation is a more complex and dynamic process than previously understood [14,16]. To date, only a single study has described such an event: a deletion DVG derived from segment 3 of equine influenza virus that contained a 30-nucleotide insert from segment 1 at the breakpoint junction [29]. This isolated example has not been widely followed up, and its biological relevance remains uncertain.

To comprehensively characterise the full diversity of DVGs in IAV, influenza A/Puerto Rico/8/1934 (H1N1) was serially passaged in embryonated chicken eggs, and viral RNA was sequenced using the Illumina NovaSeq platform. Recombination events were identified and mapped across all eight genome segments using the ViReMa pipeline, revealing patterns of DVG distribution and uncovering novel forms of segmental recombination.

## 2. Materials and Methods

### 2.1. Propagation of Influenza A PR8

Influenza A/Puerto Rico/8/1934 (H1N1) (influenza A/PR8) virus was diluted in sterile 1× PBS containing 100 μg/mL penicillin–streptomycin. Ten-day-old embryonated chicken eggs were inoculated in the allantoic cavity with 100 μL of virus dilution and incubated at 37 °C for 48 h. Embryonated eggs were then cooled to 4 °C overnight. Allantoic fluid was harvested and clarified by centrifugation (1000× *g*, 10 min, 4 °C), and supernatants were stored at −80 °C.

### 2.2. Egg Infectious Dose 50% Endpoint Assay (EID_50_)

To determine the infectious titre of influenza A/PR8, viral stocks were subjected to a 10-fold serial dilution. For each dilution, six embryonated chicken eggs were inoculated and incubated as described in ‘Propagation of Influenza A/PR8’. Viral infection was assessed in each egg using a haemagglutination assay [30]. The proportion of infected versus uninfected eggs at each dilution was used to calculate the 50% egg infectious dose (EID_50_) using the Reed and Muench method [31].

### 2.3. RNA Extraction

Viral RNA was extracted using TRIzol™ Reagent (ThermoFisher Scientific, Carlsbad, CA, USA) according to the manufacturer’s instructions, using a 5:1 ratio of TRIzol to clarified allantoic fluid. RNA pellets were resuspended in 20 μL of nuclease-free water.

### 2.4. Illumina Sequencing and Analysis

Illumina libraries were prepared and sequenced by Genewiz (Leipzig, Germany) using 150 nt paired-end reads on a NovaSeq platform. Reads were interleaved with the reformat tool (BBMap suite) and aligned to the Influenza A/PR8 genome using ViReMa v0.25 [24] with default settings. The reference genome comprised the following NCBI accessions: Segment 1 (PB2) (NC_002023), Segment 2 (PB1) (NC_002021), Segment 3 (PA) (NC_002022), Segment 4 (HA) (NC_002017), Segment 5 (NP) (NC_002019), Segment 6 (NA) (NC_002018), Segment 7 (M) (NC_002016), and Segment 8 (NS) (NC_002020). Alignment files were compressed and sorted using samtools, and per-base coverage for each segment was computed using bedtools genomecov. To normalise the read counts of multisegment DVGs, first, the mean per-segment sequencing depth (x) was calculated for all eight segments. Subsequently, for each given multisegment DVG which contained alignments to segments ‘a’ and ‘b’, the proportion of the DVG’s sequence comprised by each segment was calculated and then the normalised read count was produced by the following equation:

Normalised read count = read count ÷ ((Proportion_A_ × x_A_) + (Proportion_B_ × x_B_)),

For each DVG type, total DVG read counts represent the sum of the number of reads containing DVG junctions of this type, while unique DVG species were defined based on the distinct junction coordinates detected in the dataset. Thus, unique DVG species indicate the total number of different junction sites found for each recombination type.

### 2.5. Virus Concentration and Sucrose Gradient Purification

To isolate influenza virions, 10 mL of harvested allantoic fluid was clarified by centrifugation at 4750× *g* for 30 min at 4 °C. The supernatant was collected, and the virus was pelleted by ultracentrifugation at 39,000× *g* for 4 h at 4 °C (TH-641 rotor, ThermoFisher Scientific). The pellet was resuspended in 1 mL of sterile 1× PBS overnight at 4 °C and loaded onto a linear sucrose gradient (30%, 40%, and 55% *w*/*w*). Gradients were centrifuged at 90,000× *g* for 16 h at 4 °C. Virus-containing fractions were collected and identified by haemagglutination (HA) assay, then diluted in sterile 1x PBS to a final volume of 5 mL and ultracentrifuged at 131,000× *g* for 3 h to remove residual sucrose. Final virus pellets were resuspended in 500 μL of sterile 1× PBS overnight at 4 °C, aliquoted, and stored at −80 °C. RNA was extracted as previously described.

### 2.6. cDNA Synthesis

cDNA synthesis of viral RNA was performed using the M-MLV Reverse Transcriptase kit (Invitrogen, Carlsbad, CA, USA) with random hexamers (Invitrogen, Carlsbad, CA, USA), following the manufacturer’s instructions. cDNA was stored at −20 °C until further use.

### 2.7. RT-PCR of Multisegment DVGs

Target regions of the influenza A genome were amplified using Phusion High-Fidelity DNA Polymerase (ThermoFisher Scientific, Vilnius, Lithuania) under the following cycling conditions: initial denaturation at 98 °C for 30 s; 30 cycles of 98 °C for 10 s, 55 °C for 30 s, and 72 °C for 60 s; and a final extension at 72 °C for 10 min. Primers used for multisegment DVG detection in influenza A/PR8 are listed in Table A1. PCR products were analysed on a 2% (*w*/*v*) TAE agarose gel, compared to a GenRuler 100bp ladder (ThermoFisher Scientific, Vilnius, Lithuania), and purified using the QIAquick PCR Purification Kit (QIAGEN, Hilden, Germany).

### 2.8. DNA Cloning

PCR products and pcDNA3.1(+) vector were digested with FastDigest KpnI and NotI (ThermoFisher Scientific, Vilnius, Lithuania) at 37 °C for 1 h. PCR products without restriction sites were cloned into pCR2.1 using the TA Cloning Kit (Thermo Fisher Scientific, Carlsbad, CA, USA). Fragments <100 nt were purified using the QIAquick PCR Purification Kit (QIAGEN, Hilden, Germany); fragments >100 nt were gel-excised and purified using the QIAquick Gel Extraction Kit (QIAGEN, Hilden, Germany). Ligations were performed at a 3:1 molar ratio using T4 DNA ligase (Thermo Fisher Scientific, Carlsbad, CA, USA) and incubated at 22 °C for 1 h. A total of 2 μL of ligation product was added to 32 μL of chemically competent *E. coli* DH5α cells, incubated on ice for 30 min, and heat shocked at 42 °C for 42 s. Following the addition of 166 μL S.O.C. medium, samples were incubated at 37 °C with shaking (200 rpm) for 1 h. Transformants were plated on LB agar containing 100 μg/mL ampicillin and incubated overnight at 37 °C. Individual colonies were cultured in LB broth with ampicillin (100 μg/mL) overnight at 37 °C, 200 rpm. Plasmid DNA was extracted using the QIAGEN Plasmid Mini Kit (QIAGEN, Hilden, Germany).

### 2.9. Sanger Sequencing

Plasmid DNA was sequenced by Eurofins Genomics (Cologne, Germany) using CMV forward (5′-CGCAAATGGGCGGTAGGCGTG-3′) and BGH reverse (5′-TAGAAGGCACAGTCGAGG-3′) primers for pcDNA3.1(+) constructs. For pCR2.1 constructs, M13 forward (-20) (5′-GTAAAACGACGGCCAG-3′) and M13 reverse (5′-CAGGAAACAGCTATGAC-3′) primers were used. Sanger sequencing reads were aligned to the influenza A/PR8 reference genome using BLAST (NCBI v2.16.0+) to confirm the recombinant of multisegment DVGs.

## 3. Results

### 3.1. DVG Recombination Events Detected by ViReMa

**Figure 1:**
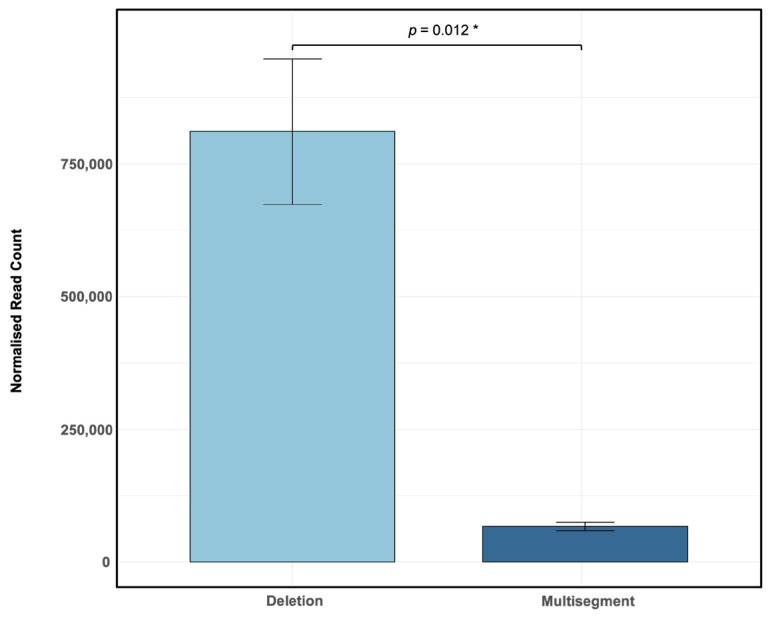
DVG recombination events identified by ViReMa analysis of Illumina sequencing data from Influenza A/PR8 infections. Embryonated chicken eggs were inoculated with a stock of influenza A/PR8 virus that had been diluted by a factor of 10^−6^. Bars represent the mean ± standard deviation (SD) of normalised junction read counts (reads per million ×) across three biological replicates (N = 3), each with three technical replicates derived from independent sequencing runs of the same sample. Statistical comparison between deletion and multisegment recombination events was performed using a paired *t*-test (df = 2.000, *p* = 0.012 *).

Influenza A/PR8 virus was propagated in embryonated chicken eggs for two passages to promote the enrichment of defective viral genomes (DVGs). RNA extracted from harvested allantoic fluid was sequenced using 150 bp paired-end reads on the Illumina NovaSeq platform. Sequencing reads were aligned to the influenza A/PR8 reference genome using ViReMa, identifying recombinant genomes characterised by deletions and multisegment recombination events. Analysis of normalised sequencing reads revealed a clear predominance of deletions (mean: 810,956 normalised reads per million) compared to multisegment events (mean: 66,968 normalised reads per million) (Figure 1), with significantly more deletions than multisegment recombination events (*p* = 0.012 *). These findings indicate that deletions are the major recombinant species generated under these experimental conditions, whereas multisegment DVGs occur at substantially lower frequencies, highlighting distinct differences in recombination type prevalence within influenza A/PR8 populations. Importantly, this is the first report to describe a large number of distinct multisegment DVGs in influenza, with an average of 8215 (SD = 352.84) unique multisegment DVG species and 22,169 (SD = 3830.86) unique deletion DVG species detected in this sample.

### 3.2. Frequency of Recombination Events Across Different Segments of Influenza A PR8

**Figure 2:**
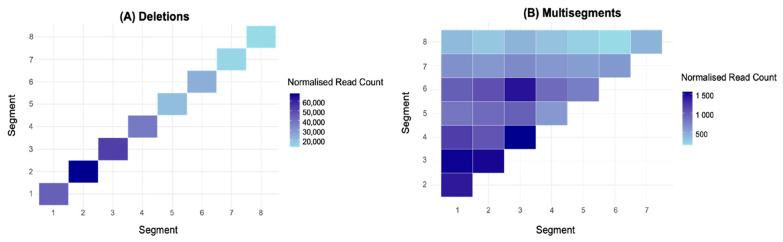
Heatmaps representing the frequency of recombination events across the eight segments of influenza A/PR8: (**A**) deletion DVGs and (**B**) multisegment DVGs. Embryonated chicken eggs were inoculated with a stock of influenza A/PR8 virus diluted 10^−6^. The data represent the mean of three biological replicates (N = 3), each with three technical replicates derived from independent sequencing runs of the same sample. Colour intensity corresponds to the sum of normalised junction read counts (reads per million ×). For deletion DVGs, one-way ANOVA followed by Tukey’s HSD post hoc test was performed to assess statistical differences in normalised read counts across segments. Full statistical details for deletion events are provided in Table A2. For multisegment DVGs, Welch’s *t*-test was conducted to determine significant differences in recombination frequency between polymerase-encoding segments (1–3) and segments (4–8).

To characterise the distribution of recombination events across the IAV genome, normalised sequencing reads were visualised as heatmaps across the eight genome segments (Figure 2). Distinct segment-specific enrichment patterns were observed for deletions, with no significant differences in normalised read counts among the polymerase-encoding segments (1–3) or within segments 4–8. However, deletions were significantly more abundant in the polymerase-encoding segments (1–3) compared to segments 4–8, although segment 4 did not differ significantly from segment 1 (*p* = 0.07441) (Figure 2A). Given the enrichment of deletions in polymerase-encoding segments, we further investigated whether this trend extended to multisegment recombination events. Focusing on recombination events involving segments 1–3 versus those involving segments 4–8, we found that multisegment recombination events were significantly more frequent among segments 1–3 than segments 4–8 (df = 3.217, *p* = < 0.001 ***, Welch’s *t*-test; Figure 2B). This recurrent pattern suggests a preferential recombination bias toward genome segments involved directly in viral RNA synthesis. Collectively, these findings highlight a non-uniform distribution of recombination events, implying that specific molecular mechanisms and segment-intrinsic features likely influence the generation of deletions and multisegment recombination events.

### 3.3. Multisegment DVGs Are Encapsidated Within Viral Particles

**Figure 3:**
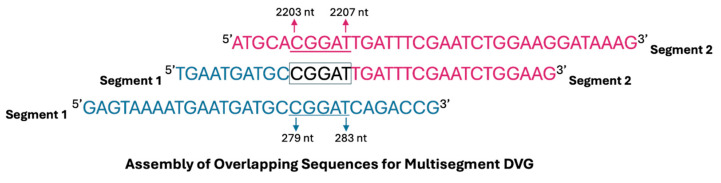
Schematic diagram illustrating the assembly of a multisegment DVG formed through recombination between segments 1 and 2 of Influenza A Virus. A short region of sequence overlap (microhomology) facilitates the junction between the two distinct segments. In this example, the junction occurs between nucleotides 279–283 of segment 1 and 2203–2207 of segment 2, resulting in a multisegment DVG with a total length of 417 nucleotides, as previously outlined in Table 1. Conserved terminal regions from segments 1 and 2 are retained, with the overlapping sequence highlighted.

To determine whether multisegment DVGs are encapsidated, sucrose density gradient centrifugation was performed to separate encapsidated from non-encapsidated viral RNA. Analysis of Illumina sequencing data revealed that multisegment DVG junctions most frequently involved segments 1, 2, and 3 of the IAV genome (Figure 2B); therefore, packaging analysis focused specifically on these polymerase-encoding segments. Viral particles were collected from the 40% sucrose fraction (representing encapsidated genomes), and RNA was extracted and reverse transcribed. To independently confirm the detection of multisegment DVGs, an RT-PCR was performed using gene-end primers targeting segments 1–3 to selectively amplify multisegment recombination events. PCR products were cloned into the pcDNA3.1(+) vector and subjected to Sanger sequencing, confirming the presence of multisegment DVGs within the encapsidated RNA population (Table 1). These findings indicate that multisegment DVGs are incorporated into viral particles rather than existing as free RNA fragments. Furthermore, most multisegment DVGs exhibited short stretches of sequence similarity (microhomology) at their junction sites (Figure 3), suggesting that recombination events may be facilitated by short homologous sequences between genome segments.

### 3.4. Persistence of Multisegment DVGs During Serial Passaging in Embryonated Chicken Eggs

**Figure 4:**
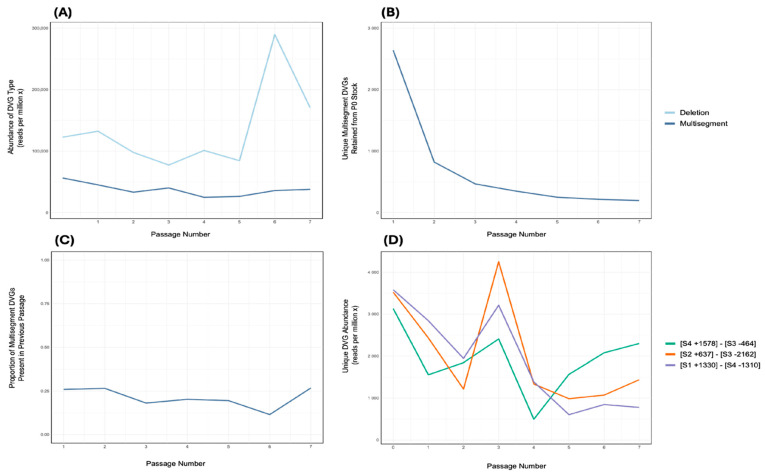
Persistence of multisegment DVGs during serial passaging of Influenza A Virus in embryonated chicken eggs. An IAV stock (Stock 6) was serially passaged seven times in embryonated chicken eggs, with the virus diluted 10^−6^ from the previous passage at each step. (**A**) The number of normalised reads that contained a deletion junction and the number of normalised reads containing a multisegment junction at each of the passage of the IAV stock. (**B**) The number of unique multisegment DVGs from the original Stock 6 (passage 0) that persisted at each subsequent passage. (**C**) The proportion of multisegment DVGs at each passage that were also detected in the preceding passage. (**D**) The abundances of three unique multisegment DVGs (represented by different colours) were selected based on being the most abundant multisegment species in at least one passage amongst the retained mulitsegment DVGs. Two of these DVGs, [S4 +1578]–[S3 −464] (green) and [S1 +1330]–[S4 −1310] (purple), showed significant correlations with the overall abundance of multisegment DVGs shown in (**A**) (Pearson’s *R*^2^ = 0.6255 and 0.5864; *p* = 0.0194 and 0.0268, respectively).

Having established the successful generation and packaging of multisegment DVGs, an independent stock of Influenza A/Puerto Rico/8/1934 (H1N1) (Stock 6) was serially passaged seven times in embryonated chicken eggs at a 10^−6^ dilution directly from the previous passage. RNA was extracted from each passage and sequenced by Illumina. Multisegment DVGs were identified using ViReMa and were consistently detected at low abundance across passages, with no apparent correlation to the abundance of DVG deletions (Figure 4A). Multisegment DVG species present in the seed stock were tracked longitudinally. Of the 11,007 distinct species detected in the seed, all but 197 were lost over the course of passaging (Figure 4B). Nonetheless, at each passage, 11.5% to 26.8% of multisegment DVG species were retained from the inoculum used to generate that passage (Figure 4C). Analysis of the most abundant DVGs retained across all seven passages revealed that, in two of three cases, their abundance was correlated with that of the overall population of multisegment DVGs (Figure 4D).

### 3.5. Dominance of a Unique Multisegment DVG and Enrichment During Passaging

**Figure 5:**
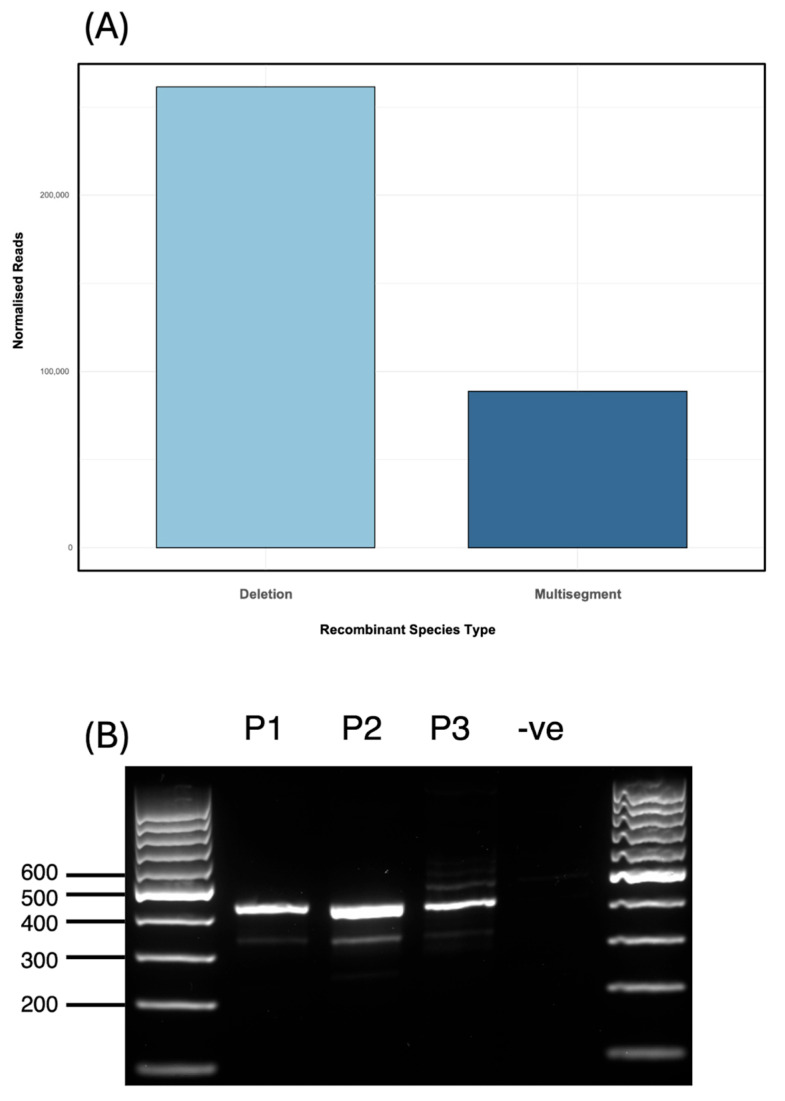
Enrichment and persistence of a dominant multisegment DVG in Stock 4. (**A**) Stock 4 was enriched in multisegment recombination events relative to earlier samples (see Figure 1), with a multisegment-to-deletion read ratio of approximately 1:3 (junction reads per million ×), in contrast to 1:12 in Figure 1. (**B**) RT-PCR validation of the S4-2 DVG multisegment recombination event between segments 4 and 2 using primers flanking the predicted junction site. The amplicon was detected in passage 0 (P0), passage 1 (P1), and passage 2 (P2), but not in the negative control of an IAV PR8 stock known to not contain S4-2 DVG (via Illumina sequencing). Ladder: GeneRuler 100 bp DNA ladder.

Sequencing of RNA from an additional independently generated IAV PR8 stock (Stock 4) revealed a DVG population notably enriched in multisegment recombination events. Normalised read counts indicated a ratio of approximately 3:1 for deletion to multisegment DVGs (deletions: 261,457; multisegments: 88,794; Figure 5A), in contrast to the ~12:1 ratio observed in the population profile shown in Figure 1. This enrichment was driven by a single dominant multisegment DVG (hereafter referred to as S4-2 DVG), which represented the most abundant recombinant junction detected, surpassing the abundance of all DVG deletion junctions. The S4-2 DVG corresponded to a recombination event between segment 4 (nucleotides 1–1700) and segment 2 (nucleotides 2107–2341) (Table 2). To confirm the presence of this specific recombination event, a 420 bp amplicon spanning the junction site of S4-2 DVG was generated by RT-PCR using DVG-specific primers flanking the predicted recombination site. The resulting PCR product was cloned and confirmed by Sanger sequencing (Table A3). The minor discrepancies between the Sanger-validated breakpoints and ViReMa predictions likely reflect the presence of microhomology (Figure 3). These short overlapping sequences can obscure exact junction sites in Sanger data, where overlapping bases may be interpreted as a range rather than a precise coordinate. In contrast, ViReMa assigns specific nucleotide positions, even within microhomologous regions. Therefore, the observed differences likely arise from this technical distinction rather than true biological variation. To investigate the persistence of the dominant S4-2 multisegment DVG, the virus Stock 4 (P0) was diluted to 10^7^ EID_50_/mL and passaged for 48 h to generate passage 1 (P1). RT-PCR analysis of RNA from P1 confirmed the presence of the same DVG band. P1 was then diluted by 10^−6^, and this dilution was used to generate passage 2 (P2). The presence of IAV was confirmed in all samples by haemagglutination (HA) assay and the S4-2 DVG was shown to be retained in both P1 and P2 (Figure 5B).

## 4. Discussion

This study aimed to investigate the diversity, segmental distribution, and encapsidation of novel defective viral genomes (DVGs) generated during the serial passaging of influenza A/PR8 virus in embryonated chicken eggs, with a particular focus on the previously uncharacterised class of multisegment DVGs. Previous studies have largely concentrated on deletion DVGs, and, although a single multisegment recombination event between segments 1 and 3 was reported more than 25 years ago [29], the existence of multisegment DVGs has remained unexplored in influenza A virus (IAV). Our analysis, using next-generation sequencing (NGS) and ViReMa, identified a broad range of multisegment DVGs, providing new evidence for the generation, packaging, and maintenance of these complex recombination events.

While deletions were the most abundant recombination events detected in the majority of NGS samples analysed, multisegment DVGs were nonetheless detected in every sample (Figure 1 and Figure 4A). This pattern is consistent with previous reports identifying deletions as the dominant DVG species during IAV replication [27,32]. However, in one independent viral stock, the S4-2 multisegment DVG became the dominant recombinant species detected (Table 2), suggesting that, occasionally, multisegment DVGs can reach high frequencies comparable to or even exceeding those of deletion DVGs.

Sucrose gradient purification confirmed that multisegment DVGs are encapsidated within viral particles rather than existing as free RNA fragments or unpackaged ribonucleoproteins (Table 1). The retention of conserved terminal sequences and packaging signals from both parental segments likely facilitates their encapsidation, consistent with previous findings that found that untranslated regions (UTRs) are essential for the efficient packaging of IAV RNA segments [33,34]. Unlike simple deletion DVGs, which retain segment-specific packaging signals and can replace their corresponding wild-type segment without disrupting the overall genome structure [10,35], multisegment DVGs combine the 5′ end of one segment with the 3′ end of another, forming chimeric RNAs. Although we have shown that multisegment DVGs are packaged into viral particles, it remains unclear which, if any, segments they replace. Their atypical structure may interfere with genome packaging fidelity by disrupting packaging signals derived from distinct segments. Together, these findings raise important questions about how the co-acquisition of mismatched packaging signals might influence genome assembly and viral fitness, warranting further investigation.

Previous studies have used serial passaging and NGS to investigate the dynamics of DVGs, with Pelz et al. [28] showing that deletion DVGs can persist and rise to dominance within IAV populations. Across seven serial passages (Figure 4A), no multisegment DVG species rose above the low baseline levels at which they were consistently detected. However, sequencing of an independent IAV stock showed a unique recombination between segments 4 and 2, which had spontaneously become the most abundant recombinant species (Figure 5; Table 2). It remains unclear whether multisegment DVGs possess intrinsic features that promote their propagation, as has been suggested for deletion DVGs [28]. Notably, the most abundant multisegment DVG detected was 1934 nt in length, and previous studies have reported that longer DVGs may be preferentially packaged [10]. Collectively, these findings suggest that, although deletion DVGs typically dominate in abundance, multisegment DVGs are capable of persisting and, under certain conditions, can rise to high frequencies. While their precise biological roles remain undefined, the fact that multisegment DVGs are retained through serial passage suggests that co-infection with wild-type virus occurs during infection. Upon co-infection, multisegment DVGs may interfere with viral replication through mechanisms previously described for classical deletion DVGs and copy-back DVGs [17]. These include replicative interference, in which DVGs compete with wild-type segments for replication resources, and stimulation of the host antiviral response via recognition by RIG-I, MDA5, or other pattern recognition receptors [11,18,19,36]. In IAV, deletion DVGs have been associated with enhanced ISG expression, potentially through IFN-independent pathways [37], while copy-back DVGs in Sendai virus strongly stimulate RIG-I-mediated antiviral signalling [36]. The persistence and occasional dominance of multisegment DVGs in viral populations indicate that they may reach levels sufficient to influence infection dynamics through one or both of these mechanisms. Future experiments comparing the yield from samples naturally enriched in multisegment DVGs with non-enriched controls would help to evaluate this [38]. Alternatively, a reverse genetics approach could be adapted to generate homogeneous multisegment DVG stocks, enabling a more controlled assessment of their biological activity [10,12,28].

The observation that multisegment DVGs often contain short stretches of microhomology at their recombination junctions supports the hypothesis that these events may arise through microhomology-guided template-switching mechanisms (Figure 3). Gribble et al. [39] demonstrated that β-coronaviruses, including MHV, MERS-CoV, and SARS-CoV-2, frequently undergo recombination during replication, with junction sites more often containing 2–10 homologous nucleotides than would be expected by chance. Their findings support a model in which recombination is facilitated by short regions of sequence homology. Consistent with this, small stretches of microhomology were observed at the junctions of multisegment DVGs identified in this study (Table 2), suggesting that similar mechanisms may operate in IAV. One plausible model is that, during multisegment DVG formation, the nascent RNA strand separates from its original template and anneals to a new segment via a region of microhomology, enabling the viral polymerase to resume replication on the newly acquired template. Although direct mechanistic evidence in IAV is limited, such a process could also underlie the formation of other DVG types, including deletions, suggesting a potentially shared recombination mechanism during viral replication.

An additional key finding was the non-uniform distribution of recombination events across the IAV genome. Both deletion and multisegment DVGs were disproportionately enriched in the polymerase-encoding segments (1–3) (Figure 2), consistent with previous IAV deletion DVG studies [15,28]. Statistical analysis confirmed significantly higher deletion frequencies in segments 1–3 compared to segments 4–8, and multisegment recombination events were likewise more frequent among polymerase-encoding segments (*p* ≤ 0.001). This segmental bias suggests that structural or functional features of polymerase segments, such as RNA secondary structures or bundling signals involved in intersegment RNA–RNA interactions, may promote recombination [35]. Similar mechanisms have been suggested to exist in SARS-CoV-2, where long-range RNA–RNA interactions are thought to influence recombination site selection [23]. In IAV, such interactions may similarly facilitate the formation of multisegment DVGs by bringing potential reinitiation sites into closer spatial proximity with the existing template strand and associated replication complex [40]. Additionally, the occurrence of recombination between different genome segments raises the possibility that, during co-infection, homologous segments from distinct viral strains (e.g., H1N1 and H3N2, or human and avian strains like H5N1) could recombine, potentially generating novel genetic variants.

Further support for the biological relevance of multisegment recombination has been provided by studies of Flock House virus (FHV). Jaworski and Routh [16] identified inter-RNA recombination between two genomic segments using ClickSeq, a low-artefact sequencing method, and confirmed these findings through both Illumina and Nanopore sequencing. These results argue against the possibility of sequencing artefacts and strengthen the evidence for genuine intersegment recombination in segmented RNA viruses. Together with the findings presented here, this highlights that intersegment recombination can occur naturally and generate complex recombinant species.

## 5. Conclusions

Defective viral genomes (DVGs) are well-established products of aberrant replication, and this study identifies a previously unrecognised class of DVGs arising from intersegment recombination. While deletion DVGs have been proposed to form through premature termination and reinitiation on the same template strand, our findings provide the first evidence that the influenza A virus (IAV) replication complex can switch templates entirely. In this model, the polymerase detaches from one genomic segment and resumes synthesis on another while retaining the nascent strand. Molecular validation confirmed that these multisegment DVGs are genuine and not sequencing artefacts. Their repeated detection across multiple independent samples indicates that this mechanism occurs frequently during IAV replication in embryonated chicken eggs. The discovery of segment-specific recombination patterns, combined with the encapsidation and persistence of multisegment DVGs through serial passage, reveals an underappreciated layer of genomic complexity in IAV populations. These findings expand the known diversity of replication-derived genome products and suggest that template switching across segments is a biologically relevant process. Future studies should focus on the molecular drivers of segmental recombination and explore the functional consequences of multisegment DVGs during infection.

## Figures and Tables

**Table 1:** Summary of Sanger sequencing results of multisegment DVG RT-PCR products isolated from sucrose density gradient fractions ^1^.

Segments of Origin	Range of 5′ Breakpoint (nts)	Range of 3′ Breakpoint (nts)	Full Length of DVG (nt)	Sample
1 and 2	279–283	2203–2207	417	1
3 and 2	101–108	1965–1972	477	
2 and 3	111–113	1742–1744	602	
3 and 1	166–167	2201–2202	306	
1 and 2	279–283	2203–2207	417	2
3 and 2	161	1819	684	
3 and 1	97–100	1976–1979	466	
1 and 2	483–488	2258–2263	566	
3 and 2	690–699	2121–2130	910	3

^1^ Three independent influenza A/PR8 samples of distinct origins were each diluted 10^−6^ and serially passaged in embryonated chicken eggs: sample 1 for 4 passages, sample 2 for 10 passages, and sample 3 for 14 passages. Sanger sequencing reads generated from cloned RT-PCR products were aligned to the influenza A/PR8 reference genome using BLAST to identify and determine the junction sites of multisegment DVGs. Most multisegment DVGs contain a central deletion and exhibit short regions of sequence overlap (microhomology) at the junctions between different genome segments, represented by the breakpoint range. For these samples, a total of 25 clones were sequenced by Sanger sequencing, of which 9 positive clones were confirmed (4/12 for sample 1, 4/8 for sample 2, and 1/5 for sample 3). Corresponding raw Sanger sequencing results are provided in Table A3.

**Table 2:** Summary of the five most abundant unique DVG junctions (based on read count).

Segment of Origin (5′)	Segment of Origin (3′)	5′ Breakpoint	3′ Breakpoint	Type of DVG	Normalised Reads
4	2	1700	2107	Multisegment	54,695.19
3	3	137	2074	Deletion	18,372.05
4	4	97	1636	Deletion	15,295.15
3	3	470	474	Deletion	14,100.86
2	4	158	1972	Multisegment	12,592.70

## Data Availability

The data supporting the findings of this study are available within the article and its Supplementary Materials. Raw Sanger sequencing files are provided in Supplementary File S1; Illumina sequencing data are not publicly available due to restrictions imposed by the study funder, OVO Biomanufacturing, but may be made available by the corresponding author upon reasonable request and with permission from OVO Biomanufacturing.

## Supplementary Materials

The following supporting information can be downloaded at: https://www.mdpi.com/article/10.3390/v17060856/s1. Supplementary File S1 contains raw Sanger sequencing chromatograms (.ab1 files) for multisegment DVG junctions.

## Abbreviations

The following abbreviations are used in this manuscript:

IAV	Influenza A Virus
DVG	Defective Viral Genome
DIP	Defective Interfering Particle
HA	Haemagglutinin
NA	Neuraminidase
NP	Nucleoprotein
PB1	Polymerase Basic 1
PB2	Polymerase Basic 2
PA	Polymerase Acidic
NS	Non-Structural Segment
M	Matrix
IFN	Interferon
RIG-I	Retinoic Acid-Inducible Gene I
MDA5	Melanoma Differentiation-Associated Protein 5
NGS	Next-Generation Sequencing
RT-PCR	Reverse Transcription Polymerase Chain Reaction
cDNA	Complementary DNA
EID_50_	Egg Infectious Dose 50%
NCBI	National Center for Biotechnology Information
Nt	Nucleotide
SD	Standard Deviation

## Appendix A

**Table A1:** Primers for multisegment DVGs.

Primer	Primer Sequence (5′ to 3′)
Segment 1 Forward	**GATCAT**GGTACCCGCAGTCTCGCACCCGCGA
Segment 1 Reverse	**ATGATC**GCGGCCGCGCTGTCTGGCTGTCAGTAAG
Segment 2 Forward	**GATCAT**GGTACCGCTATAAGCACAACTTTCCCTTATACC
Segment 2 Reverse	**ATGATC**GCGGCCGCCTAAATTCACTATTTTTGCCGTCTGAG
Segment 3 Forward	**GATCAT**GGTACCCCGATGATTGTCGAGCTTGCG
Segment 3 Reverse	**ATGATC**GCGGCCGCGGACAGTATGGATAGCAAATAG
Segment 4 Forward	GAGTTCTACCACAAGTGTGACAATG

**(Bold)** Random sequence upstream of the restriction site. (Underlined) Restriction enzyme sequences: Kpn1, GGTACC; Not1, GCGGCCGCC.

**Table A2:** Tukey post hoc test results comparing normalised deletion DVG read counts between genome segments.

Segment Comparison	*p*-Value
2-1	0.425
3-1	0.714
4-1	0.074
5-1	0.009 (**)
6-1	0.003 (**)
7-1	<0.001 (***)
8-1	<0.001 (***)
3-2	1.000
4-2	0.001 (**)
5-2	<0.001 (***)
6-2	<0.001 (***)
7-2	<0.001 (***)
8-2	<0.001 (***)
4-3	0.003 (**)
5-3	<0.001 (***)
6-3	<0.001 (***)
7-3	<0.001 (***)
8-3	<0.001 (***)
5-4	0.951
6-4	0.724
7-4	0.163
8-4	0.055
6-5	0.999
7-5	0.678
8-5	0.338
7-6	0.931
8-6	0.645
8-7	0.988

Pairwise comparisons were performed following a significant one-way ANOVA across segments 1–8. The table shows the segment pairs tested and the corresponding *p*-values. Asterisks indicate levels of statistical significance: *p* < 0.01 (**), *p* < 0.001 (***).

**Table A3:** Sanger sequence file key for multisegment DVGs.

Segments of Origin	Predicted Full Length of DVG (nt)	Sanger Sequencing File ID	Sample No.
1 and 2	417	EIN537/EIN536	1
3 and 2	477	EIN541/EIN540	
2 and 3	602	EIN539/EIN544	
3 and 1	306	EIN547/EIN546	
1 and 2	417	HAH679/HAH678	2
3 and 2	684	HAH695/HAH694	
3 and 1	466	HAH697/HAH696	
1 and 2	566	HAH701/HAH700	
3 and 2	910	HQZ427	3
4 and 2	1934	HNS027/HNS026	4

This table lists the multisegment DVG samples subjected to Sanger sequencing, including file names, corresponding sample identifiers, and key junction characteristics. Supplementary File S1 contains raw Sanger sequencing chromatograms (.ab1 files) for multisegment DVG junctions. These files contain original chromatogram files (.ab1 format) generated by the Sanger sequencing of multisegment defective viral genomes (DVGs).
